# Pharmacology and Adverse Events of Emergency-Use Authorized Medication in Moderate to Severe COVID-19

**DOI:** 10.3390/ph14100955

**Published:** 2021-09-23

**Authors:** Jen-Yu Hsu, Yan-Chiao Mao, Po-Yu Liu, Kuo-Lung Lai

**Affiliations:** 1 Department of Internal Medicine, Division of Infectious Diseases, Taichung Veterans General Hospital, Taichung 407219, Taiwan; b94401012@g.ntu.edu.tw; 2 Department of Emergency Medicine, Division of Clinical Toxicology, Taichung Veterans General Hospital, Taichung 407219, Taiwan; doc1385e@gmail.com; 3 National Defense Medical Center, School of Medicine, Taipei 114201, Taiwan; 4 Department of Internal Medicine, Division of Allergy, Immunology and Rheumatology, Taichung Veterans General Hospital, Taichung 407219, Taiwan

**Keywords:** adverse event, baricitinib, COVID-19, remdesivir, sarilumab, tocilizumab

## Abstract

Some effective drugs have been approved or issued an Emergency Use Authorization for the treatment of COVID-19 in hospitalized patients, but post-market surveillance is warranted to monitor adverse events. We reviewed clinical trials and case reports in patients with moderate-to-severe COVID-19 infection who received remdesivir, baricitinib, tocilizumab, or sarilumab. The drug-specific pharmacokinetics, toxicity, and drug interactions are summarized in this study. Remdesivir and baricitinib are small-molecule drugs that are mainly metabolized by the kidneys, while tocilizumab and sarilumab are monoclonal antibody drugs with metabolic pathways that are currently not fully understood. The most common adverse events of these drugs are alterations in liver function, but serious adverse events have rarely been attributed to them. Only a few studies have reported that remdesivir might be cardiotoxic and that baricitinib might cause thromboembolism. Biological agents such as baricitinib, tocilizumab, and sarilumab could inhibit the pathway of inflammatory processes, leading to immune dysregulation, so the risk of secondary infection should be assessed before prescribing. Further recognition of the pathogenic mechanism and risk factors of adverse events is essential for optimizing treatment strategies.

## 1. Introduction

As of 20 September 2021, there have been 228.4 million confirmed cases of coronavirus disease 2019 (COVID-19), which is caused by Severe Acute Respiratory Syndrome coronavirus 2 (SARS-CoV-2), and these cases include 4.7 million deaths [1]. The incidence and mortality rates were higher in certain regions and environments [2], and investigations on anti-coronavirus vaccines have been widely initiated [3]. However, for people infected with the disease, the choice of drugs is paramount. Patients without shortness of breath, dyspnea, or abnormal chest imaging are considered to have mild illness. Patients who develop dyspnea have at least moderate-severity disease, which often warrants hospitalization, and the need for oxygenation or ventilatory support indicates severe disease [4]. The U.S. Food and Drug Administration (US FDA) issued an Emergency Use Authorization (EUA) for casirivimab plus imdevimab, bamlanivimab plus etesevimab, and sotrovimab for the treatment of mild-to-moderate COVID-19 [5,6,7]. For moderate-to-severe infections, in addition to corticosteroids and emergency use of convalescent plasma [8,9], the US FDA approved or issued an EUA for remdesivir, remdesivir plus baricitinib, and tocilizumab 2020 [10,11,12].

In the development of a drug for moderate-to-severe infections, a major goal is to maximize its effectiveness while minimizing any safety issues, which is a delicate balancing act. The US FDA approved corticosteroids in July 2020 [8] and issued an EUA for emergency use of convalescent plasma in August 2020 [9] for hospitalized COVID-19 patients, but the available drugs for moderate-to-severe infections were still limited. The US FDA had issued but revoked its EUA for hydroxychloroquine and chloroquine in patients with severe COVID-19 in June 2020, noting that the known and potential toxicity exceeded expectations [13]. Lopinavir/ritonavir was also not recommended for hospitalized patients, mainly due to the lack of obvious efficacy and the high risk of drug interaction [14,15]. In COVID-19 patients treated with hydroxychloroquine, chloroquine, or lopinavir/ritonavir, the most commonly reported severe adverse event (AE) resulted from cardiotoxicity, such as QT interval prolongation. In contrast, other drugs such as remdesivir, baricitinib, tocilizumab, and sarilumab seemed to be effective but were less toxic [16,17,18].

### Chemical Structure and Molecular Properties

Remdesivir, baricitinib, tocilizumab, and sarilumab have distinct chemical structures and molecular properties [19], as shown in Table 1, which give them their own unique mechanisms of action in the treatment of COVID-19. The US FDA approved remdesivir in intravenous injection form in October 2020 [10] based on the ACTT-1 clinical trial [20], and issued an EUA for baricitinib oral tablets in combination with remdesivir in November 2020 [11], based on the ACTT-2 clinical trial [17]. In June 2021, the US FDA issued an EUA for tocilizumab in intravenous injection form [12], based on the RECOVERY, EMPACTA, COVACTA, and REMDACTA clinical trials [21,22,23,24]. Sarilumab might have therapeutic effects, but research is still ongoing [18,25,26,27,28,29]. The completed and ongoing randomized controlled trials of these drugs are presented in Table 2. This study aimed to summarize the available evidence to address the pharmacokinetics, toxicity, and drug interactions of these four candidate drugs: remdesivir, baricitinib, tocilizumab, and sarilumab.

## 2. Literature Search Strategy

We reviewed the US FDA COVID-19 information on regulated products and consulted Micromedex^®^ Solutions Drugs Interactions as well as Lexicomp^®^ Drug Interactions for COVID-19 therapies. We conducted a search of PubMed for articles with the keywords “((remdesivir) or (baricitinib) or (tocilizumab) or (sarilumab)) and (COVID-19) and ((adverse event) or (toxicity) or (drug interaction))”. Reference lists of all identified studies were also searched. 

**Figure 1:**
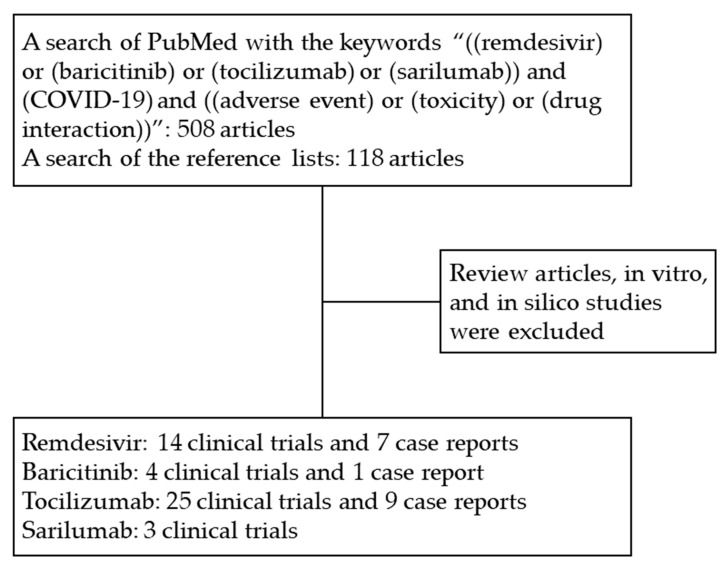
Study flow. Abbreviations: COVID-19, coronavirus disease 2019.

A total of 626 articles were collected. The clinical trials and case reports of patients with moderate-to-severe COVID-19 infection who received the targeted drugs were included in our analysis. Review articles, in vitro, and in silico studies were excluded. Articles not published in English were excluded. As shown in Figure 1, we identified 14 trials and 7 case reports on remdesivir, 4 trials and 1 case report on baricitinib, 25 trials and 9 case reports on tocilizumab, and 3 trials on sarilumab. All of the studies were conducted in America, Europe, and Asia.

## 3. Remdesivir

### 3.1. Indication

Remdesivir in intravenous form was only approved for the treatment of COVID-19 in hospitalized patients aged ≥12 years and weighing ≥40 kg [10], and was granted an EUA for hospitalized patients aged <12 years and weighing ≥3.5 kg by the US FDA [30]. Remdesivir is indicated in hospitalized patients requiring supplemental oxygen, invasive mechanical ventilation, or extracorporeal membrane oxygenation (ECMO) [30]. For patients who do not need respiratory support, remdesivir does not offer significant benefit at day 28, so its use is not recommended [31]. The Infectious Diseases Society of America (IDSA) and the National Institutes of Health (NIH) recommend that remdesivir should be used only for hospitalized patients who require non-invasive oxygen therapy [4,32], while the World Health Organization (WHO) suggests against administering remdesivir in addition to usual care [33].

### 3.2. Pharmacokinetics and Mechanism of Action

Remdesivir has moderate protein-binding capacity (88.0% to 93.6% bound) in human plasma, and the volume of distribution varies from 56.3 to 73.4 L depending on the amount of dose administered [34]. These properties make remdesivir tend to leave the plasma and enter the extravascular compartments of the body. Remdesivir, as a viral RNA-dependent RNA polymerase inhibitor, is extensively distributed in cells and metabolizes to an active nucleoside triphosphate with a half-life of 20–25 h, which competes with adenosine triphosphate for incorporation into viral RNA, causing premature chain termination and inhibition of viral replication [34,35]. It has been shown that 74% of remdesivir and its active metabolite are eliminated by the kidneys, and 18% are eliminated through feces. Remdesivir is not recommended in patients with severe renal insufficiency, but some studies have shown that the usual dose of remdesivir might be safe for patients with severe renal insufficiency [36], end-stage renal disease on hemodialysis [37], or kidney transplantation [38].

### 3.3. Recommended Dosage

The recommended dosage of remdesivir for COVID-19 patients is a single loading dose of 200 mg followed by once-daily maintenance doses of 100 mg via intravenous infusion over 30 to 120 min. Duration is generally 5 to 10 days. In this case, a high and stable intracellular concentration of active triphosphate could be achieved to effectively inhibit SARS-CoV-2 without causing serious AEs or death [34]. However, the clinically toxic dose has not been established, and the toxicity caused by acute exposure to remdesivir can only be roughly estimated from the currently reported AEs.

### 3.4. Adverse Events

Among patients receiving remdesivir treatment, the prevalence of AEs was between 51% and 74% [31,39,40]. It is unclear whether patients receiving remdesivir would have had more AEs than those receiving placebo [20,39], and it is also inconclusive as to whether patients receiving remdesivir for 10 days would have had more AEs than those receiving remdesivir for 5 days [31,40]. Grade 3 or 4 AEs occurred in 10% to 51% of patients receiving remdesivir [17,20,31,40,41], but only 5% to 15% were considered to be related to remdesivir [17,20]. The most frequently reported AEs included alterations in liver-function studies (i.e., elevated alanine aminotransferase (ALT) and aspartate aminotransferase (AST) and hyperbilirubinemia), increased serum levels of creatinine, decreased lymphocyte count, anemia, thrombocytopenia, respiratory failure, increased blood glucose level, hypokalemia, constipation, and nausea [17,20,31,39,40,41,42]. Serious AEs developed in 5% to 35% of patients receiving remdesivir [17,20,31,39,40], the most common of which was respiratory failure [17,20,39,40], but few AEs were considered to be related to remdesivir [17]. Approximately 2% to 12% of patients discontinued treatment because of AEs [20,31,39,40], but whether AEs are more likely to occur in patients receiving remdesivir remains controversial [20,39]. The proportion of COVID-19 patients who died after receiving remdesivir ranged from 1% to 15% [20,31,39,41]. The causes of death included multiple organ failure and cardiopulmonary arrest [20,39,41,43], which were mainly attributed to the worsening of COVID-19 [41]. Almost all deaths were considered unrelated to remdesivir [20,31,39,43].

In addition to abnormal liver and kidney function caused by COVID-19 infection [44,45], some patients developed abnormal liver and kidney function after receiving remdesivir [39,40]. Remdesivir-related liver dysfunction lacks a clear pathogenesis, but gradually improves after the culprit drug is discontinued [34]. Whether remdesivir can cause renal dysfunction is unclear. Few deaths have been attributed to liver or kidney failure caused by remdesivir [43]. Another AE caused by remdesivir is atrioventricular block, leading to bradycardia and cardiac arrest, which was found in patients treated for Ebola virus disease [46] and COVID-19 infection [47,48,49]. A possible explanation is that although remdesivir triphosphate has a weak ability to inhibit mammalian DNA and RNA polymerases, it might still cause subsequent mitochondrial dysfunction, resulting in cardiotoxicity [50]. Moreover, remdesivir, as a nucleotide analog that resembles adenosine triphosphate, has partial affinity for adenosine A1 receptors, which might block the atrioventricular node and thus delay the conduction [51]. Nevertheless, studies on the cardiotoxicity caused by remdesivir are still insufficient. Remdesivir has a paucity of other severe adverse reactions, but cases of anaphylaxis have been reported [52]. Remdesivir infiltration could also result in local complications due to the low pH (~4) of the nonbuffered remdesivir solution [53].

Remdesivir is a substrate of the cytochrome P450 (CYP) 2C8, CYP2D6, CYP3A4, organic anion transporting polypeptide 1B1 (OATP1B1), and P-glycoprotein (P-gp), and it is also an inhibitor of the CYP3A4, OATP1B1, OATP1B3, bile salt export pump (BSEP), multidrug resistance protein 4 (MRP4), and sodium and taurocholate cotransporting polypeptide (NTCP). However, based on rapid distribution, metabolism and clearance of remdesivir, the likelihood of clinically significant interactions is low [16], and only a few cases have been reported [54,55,56]. In contrast to cell-culture results showing that concurrent use of chloroquine or hydroxychloroquine might reduce the intracellular metabolic activation of remdesivir and lessen the antiviral activity, it was found in clinical practice that concurrent use of chloroquine and amiodarone might further inhibit the P-gp of hepatocytes that could excrete remdesivir, leading to the accumulation of remdesivir in the liver and causing the subsequent hepatotoxicity [54]. Two case reports showed that the simultaneous use of remdesivir and dexamethasone might enhance the effects of warfarin and prolong the prothrombin time [55]. Another case report showed that remdesivir might change the effectiveness of tramadol [56]. The possible role of remdesivir in drug interference has yet to be confirmed. 

## 4. Baricitinib

### 4.1. Indication

Baricitinib is a crushable oral tablet that is used in combination with methotrexate to relieve the signs and symptoms of moderate to severe rheumatoid arthritis (RA) in patients who have responded inadequately to one or more disease-modifying anti-rheumatic drugs (DMARDs) [57]. In the case that remdesivir is an effective treatment for hospitalized patients with COVID-19 based on the ACTT-1 clinical trial [20], the combined use of baricitinib seems to further improve clinical outcomes based on the ACTT-2 clinical trial [17]. Baricitinib was granted an EUA by the US FDA in combination with remdesivir for the treatment of COVID-19 in hospitalized patients aged ≥2 years requiring supplemental oxygen, invasive mechanical ventilation, or ECMO [11]. However, the IDSA and the NIH recommend that baricitinib should be used only for hospitalized patients who require non-invasive oxygen therapy [4,32].

### 4.2. Pharmacokinetics and Mechanism of Action

Baricitinib, as an inhibitor of Janus kinase (JAK), blocking the subtypes JAK1 and JAK2, in sequence blocks the pathway of cytokines such as interleukin-6 (IL-6) responsible for inflammatory processes. Moreover, baricitinib has been predicted to inhibit receptor-mediated endocytosis of SARS-CoV-2 by machine-learning algorithms [58]. The oral bioavailability of baricitinib is about 80%. Baricitinib is 50% bound to plasma proteins, and the mean volume of distribution is 76 L, showing distribution of baricitinib into tissues. Studies show that 75% and 20% of baricitinib are excreted in urine and feces, respectively, and approximately 6% of the dose is identified as metabolites. The half-life of elimination is 8 h to 12 h [59]. In patients with any level of renal impairment or moderate-to-severe hepatic impairment, decreasing the dose or avoiding the use of baricitinib should be considered.

### 4.3. Recommended Dosage

The recommended dosage of baricitinib for COVID-19 patients over 9 years old and younger than 9 years old are 4 mg and 2 mg orally once daily, respectively, for 14 days or until hospital discharge. The effect of IL-6 is maximally inhibited 1 h following oral administration of baricitinib, and it returns to near baseline by 24 h. The steady-state concentration of baricitinib is achieved within 2 to 3 days. Proportional increases of the plasma concentration were observed within the range of therapeutically permitted doses. The clinically toxic dose for the COVID-19 patients has not been established, but the use of baricitinib might be associated with serious infections, thromboembolic events, and hypersensitivity reaction, based on experience using this drug to treat other diseases [60].

### 4.4. Adverse Events

It is generally believed that COVID-19 patients using baricitinib on a short-term basis are unlikely to have serious AEs. In a clinical trial of 507 adult patients with COVID-19 who received baricitinib plus remdesivir, 207 patients (40.7%) had grade 3 or 4 AEs, of which a total of 25 AEs were related to the treatment, which was similar to the results of the 509 patients who received remdesivir alone. Serious infection, venous thromboembolism, and pulmonary embolism occurred in 6%, 4%, and 1% of patients treated with baricitinib plus remdesivir, respectively, which did not achieve statistically significant differences compared to those treated with remdesivir alone [17]. This might be partially explained by the fact that faster recovery could lower the risk of hospital-associated complications and may also be partly attributed to the use of thromboembolism prophylaxis. However, the potential benefits of treatment with baricitinib against potential risks should be weighed in patients with neutropenia, lymphopenia or other active infections, such as tuberculosis [11] and hepatitis B virus infection [61,62]. A higher dose of baricitinib might result in early stabilization of respiratory functions, but it could increase the risk of thromboembolism [63]. Baricitinib should be promptly discontinued if a serious hypersensitivity reaction occurs [57].

Baricitinib is a substrate of the CYP3A4, P-gp, breast cancer resistance protein (BCRP), organic anionic transporter (OAT) 3, and multidrug and toxic extrusion protein (MATE) 2-K transporters. CYP3A4 is the main metabolizing enzyme of baricitinib, but the clinical use of ketoconazole (CYP3A inhibitor) or rifampin (CYP3A inducer) has no effect on the pharmacokinetics of baricitinib [64]. The administration of probenecid (strong OAT3 inhibitor) could decrease the clearance of baricitinib, indicating that the dosage of baricitinib should be reduced in patients taking a strong OAT3 inhibitor concurrently, although diclofenac and ibuprofen (OAT3 inhibitors with less inhibition potential) did not appear to have any effect in this regard in silico [65]. Baricitinib is also an inhibitor of OAT1, OAT2, OAT3, OATP1B3, BCRP, MATE1, and MATE2-K, but it is unlikely to cause clinically meaningful changes.

## 5. Tocilizumab

### 5.1. Indication

Tocilizumab in subcutaneous or intravenous form is indicated for the treatment of chimeric antigen receptor T cell-induced cytokine release syndrome, active polyarticular juvenile idiopathic arthritis, systemic sclerosis-associated interstitial lung disease, moderately to severely active RA that cannot be treated with DMARDs, active systemic juvenile idiopathic arthritis, and giant cell arteritis [66]. Tocilizumab in intravenous form was granted an EUA by the US FDA for the treatment of COVID-19 in hospitalized patients aged ≥2 years receiving systemic corticosteroids and requiring supplemental oxygen, non-invasive or invasive mechanical ventilation, or ECMO [12]. The IDSA, the NIH, and the WHO recommend that tocilizumab could be used in hospitalized patients with severe or critical COVID-19 [4,32,33].

### 5.2. Pharmacokinetics and Mechanism of Action

Tocilizumab, as an IL-6 receptor inhibitor that binds specifically to both the soluble and membrane-bound IL-6 receptors, has been shown to inhibit IL-6-mediated inflammatory processes. In COVID-19 adult patients who received intravenous tocilizumab, the mean volume of distribution was 8.75 L, and the rate of clearance ranged from 17.6 to 35.4 mL per hour, increasing with the severity of the disease. The mechanism of metabolism and excretion of tocilizumab has not been clarified, but its high molecular weight of 148.0 kDa [19,67], which is 250 times and 400 times that of remdesivir and baricitinib, respectively, indicates that tocilizumab is likely not readily excreted from the kidneys. Tocilizumab is presumed to be metabolized to smaller proteins and amino acids by proteolytic enzymes. The dosage adjustments in patients with hepatic or renal impairment has yet to be determined.

### 5.3. Recommended Dosage

The recommended dosage of tocilizumab for COVID-19 patients over 30 kg and under 30 kg comprises a single loading dose of 8 mg/kg and 12 mg/kg (maximum dose 800 mg per infusion) over 60 min, respectively, plus a second dose at least 8 h after initial infusion if clinical signs or symptoms worsen or do not improve. In adult patients with COVID-19, the estimated median peak concentrations for a single dose and two doses are 151 µg/mL and 290 µg/mL, respectively, and the concentration falls below the limit of quantification by day 35. The clinically toxic dose for COVID-19 patients has not been established. 

### 5.4. Adverse Events

Among COVID-19 patients receiving tocilizumab, the prevalence of AEs was between 23% and 77% [22,23,68,69,70,71,72,73], and the prevalence of serious AEs was between 0% and 34% [18,22,23,68,69,70,71,73,74,75]. These figures were not different compared with those of patients who received a placebo [23,68,69,70]. Only about 0% to 6% of patients receiving tocilizumab were considered to have causal AEs [69,71,74,75]. The most common AE was alteration in liver-function studies (i.e., elevated ALT and AST and hyperbilirubinemia) [68,70,71,72,73,75,76,77,78], but whether AE was more likely to occur in patients receiving tocilizumab remains controversial [77,79]. Other common AEs include neutropenia [70,71,73,74,77], which appeared to occur more frequently in patients receiving tocilizumab [74,77] and might be managed with granulocyte colony-stimulating factor if necessary, and secondary bacterial infections [18,21,22,23,74,77,78,80]. There were also reports of allergic reactions [72,81], sudden cardiorespiratory collapse [68,69], acute respiratory distress syndrome [70], pulmonary embolism [77], anemia [68], thrombocytopenia [68], increased serum levels of creatinine [75], and neurological adverse effects [82]. The mortality rate was between 10% and 23% [22,23,69,72,75], but almost all deaths were not related to tocilizumab [21,72].

Tocilizumab is effective in reducing the mortality of severe COVID-19 infection, but regular use of tocilizumab might be harmful due to excessive inhibition of IL-6-mediated inflammatory immune response [83]. Other infections such as tuberculosis might pose a serious threat during the treatment with tocilizumab. It remains controversial as to whether COVID-19 patients receiving tocilizumab could have fewer secondary infections due to faster recovery [70,74,80]. Those receiving tocilizumab might not have related complications at the early stage [84], but careful monitoring for possible late-onset infections and toxicities is warranted [77,85,86]. Other disease processes may harm the gastrointestinal tract, and as tocilizumab could simply block healing of the gastrointestinal tract, ulcers or bleeding may occur [71,87].

Tocilizumab has few or no known serious drug interactions clinically [16,88,89]. The rise of IL-6 during inflammation in COVID-19 patients might inhibit the activity of CYP enzymes, thereby changing the concentration of other drugs. Tocilizumab could block the IL-6 pathway in time and normalize the metabolism, but the impact of these changes on drugs with a narrow therapeutic index remains unclear. Caution is required, especially when co-administering with myelotoxic drugs due to the potential additive toxicity [90]. Concomitant use of other immunosuppressive drugs should also be avoided because of the possibility of increased risk of infection.

## 6. Sarilumab

### 6.1. Indication

Sarilumab in subcutaneous form is indicated for the treatment of patients with moderately to severely active RA who have had an inadequate response or intolerance to DMARDs [91]. Sarilumab in subcutaneous or intravenous form is currently under investigational use for the treatment of COVID-19, and its efficacy and safety have not yet been determined [26,92,93,94]. The NIH recommends that sarilumab could be used instead of tocilizumab in hospitalized patients with severe or critical COVID-19 [4].

### 6.2. Pharmacokinetics and Mechanism of Action

Sarilumab inhibits IL-6-mediated inflammatory immune response by binding to IL-6 receptors that are both soluble and membrane-bound. Although data on the absorption, distribution, metabolism, and excretion of sarilumab are still lacking in COVID-19 patients, sarilumab has a molecular weight of approximately 150 kDa [19,91], which is similar to tocilizumab, which implies that it might not be readily excreted from the kidneys.

### 6.3. Recommended Dosage

The dosage of sarilumab under investigation was a single loading dose of 200 mg subcutaneously or 200 to 800 mg intravenously. 

### 6.4. Adverse Events

In the studies of COVID-19 patients receiving intravenous injection of sarilumab 200 to 400 mg, the percentage of AEs ranged from 55% to 80%, the percentage of serious AEs ranged from 26% to 66%, and the percentage of death ranged from 10% to 37%, none of which showed a statistically significant difference in comparison with those receiving placebo [26,94]. However, it was found that compared with patients receiving placebo, those receiving sarilumab seemed to have higher incidences of alteration in liver function studies, neutropenia, secondary infection, and allergic reaction [26,94]. Active or latent tuberculosis was found to be another issue that should be managed before and during treatment with sarilumab [91].

## 7. Conclusions

A number of drugs with potential therapeutic efficacy have been proposed for the treatment of COVID-19 patients requiring hospitalization. However, AEs are increasingly being reported. We reviewed the pharmacokinetics, toxicity, and drug interactions of four drugs: remdesivir, baricitinib, tocilizumab, and sarilumab. The information on these drugs is summarized in Table 3. The most common AE among these drugs was alteration in liver-function studies, but serious AEs have rarely been attributed to them. Remdesivir might cause cardiotoxicity such as atrioventricular block, so close cardiac monitoring is essential. Patients receiving baricitinib might have a higher risk of thromboembolism, and thus prophylaxis should be considered. When IL-6-mediated inflammatory processes are inhibited by biological agents such as baricitinib, tocilizumab, and sarilumab, immune dysregulation seems to be another important issue, and thus the potential benefits of treatment should be evaluated against the possible increased risks of secondary infection. Although AEs could resolve spontaneously in most patients after discontinuing the culprit drug, greater vigilance is warranted for patients with high susceptibility in order to prevent irreversible damage. Further study is needed to explore the pathogenic mechanisms involved and to determine the relevant risk factors so that novel treatment strategies can be developed.

## Figures and Tables

**Table 1:** The chemical structures and molecular properties of available drugs in moderate-to-severe COVID-19.

Drugs	Type	Chemical Formula	Average Weight	US FDA Labeled Indication Other Than COVID-19	Possible Mechanisms in Treatment of COVID-19
Remdesivir	Small Molecule	C_27_H_35_N_6_O_8_P	602.6 Da	None	The nucleotide analogue inhibit viral nucleotide synthesis to stop viral replication.
Baricitinib	Small Molecule	C_16_H_17_N_7_O_2_S	371.4 Da	1. Rheumatoid arthritis (moderate to severe)	The JAK inhibitor blocks JAK-STAT signaling pathway and inflammatory response.
Tocilizumab	Monoclonal antibody	C_6428_H_9976_N_1720_O_2018_S_42_	148,000.0 Da	1. Cytokine release syndrome 2. Juvenile rheumatoid arthritis 3. Lung disease with systemic sclerosis 4. Rheumatoid arthritis (moderate to severe) 5. Systemic onset juvenile chronic arthritis 6. Temporal arteritis	The IL-6 receptor antagonist inhibit IL-6 signaling pathway and inflammatory response.
Sarilumab	Monoclonal antibody	C_6388_H_9918_N_1718_O_1998_S_44_	150,000.0 Da	1. Rheumatoid arthritis (moderate to severe)	The IL-6 receptor antagonist inhibit IL-6 signaling pathway and inflammatory response.

Abbreviations: COVID-19, coronavirus disease 2019; Da, dalton; IL-6, Interleukin-6; JAK, Janus kinase; STAT, signal transducer and activator of transcription; US FDA, U.S. Food and Drug Administration.

**Table 2:** The completed and ongoing randomized controlled trials of available drugs in moderate-to-severe COVID-19.

Drugs	Trial Names	Trial Sites	Inclusion Criteria	Patient Numbers	Dosage	Efficacy	Safety
Remdesivir	ACTT-1 [20]	The United States (45 sites), Denmark (8), the United Kingdom (5), Greece (4), Germany (3), Korea (2), Mexico (2), Spain (2), Japan (1), and Singapore (1)	Adults who were hospitalized with COVID-19 and had evidence of lower respiratory tract infection	541 assigned to the remdesivir group and 521 to the placebo group	Remdesivir was administered intravenously as a 200-mg loading dose on day 1, followed by a 100-mg maintenance dose administered daily on days 2 through 10 or until hospital discharge or death	Remdesivir was superior to placebo in shortening the time to recovery; the mortality rates were 6.7% with remdesivir and 11.9% with placebo by day 15 (HR, 0.55; 95% CI, 0.36 to 0.83) and 11.4% with remdesivir and 15.2% with placebo by day 29 (HR, 0.73; 95% CI, 0.52 to 1.03)	Serious adverse events were reported in 131 of the 532 patients who received remdesivir (24.6%) and in 163 of the 516 patients who received placebo (31.6%)
Baricitinib	ACTT-2 [17]	The United States (55 sites), Singapore (4), South Korea (2), Mexico (2), Japan (1), Spain (1), the United Kingdom (1), and Denmark (1)	Adults with COVID-19 who received remdesivir (≤10 days) and either baricitinib (≤14 days) or placebo (control)	515 assigned to combination treatment and 518 to control	Baricitinib was administered as a 4-mg daily dose for 14 days or until hospital discharge.	Baricitinib plus remdesivir was superior to remdesivir alone in reducing recovery time; the 28-day mortality was 5.1% in the combination group and 7.8% in the control group (HR, 0.65; 95% CI, 0.39 to 1.09)	Serious adverse events were less frequent in the combination group than in the control group (16.0% vs. 21.0%), as were new infections (5.9% vs. 11.2%), under the recommendation of venous thromboembolism prophylaxis
Tocilizumab	RECOVERY [21]	The United Kingdom	Adults with COVID-19 who had hypoxia (oxygen saturation <92% on air or requiring oxygen therapy) and evidence of systemic inflammation (C-reactive protein >=75 mg/L)	2022 randomly allocated to tocilizumab and 2094 to usual care	Tocilizumab was given intravenously as a dose of 400–800 mg, followed by either a second dose 12–24 h later or not	Tocilizumab was superior to placebo in discharge from hospital within 28 days (57% vs. 50%); patients allocated tocilizumab were less likely to reach the composite endpoint of invasive mechanical ventilation or death (35% vs. 42%; RR 0.84; 95% CI 0.77–0.92)	There were three reports of serious adverse reactions believed to be related to tocilizumab: one each of otitis externa, Staphylococcus aureus bacteremia, and lung abscess, all of which resolved with standard treatment
Tocilizumab	EMPACTA [22]	The United States (45 sites), Brazil (6), Peru (5), South Africa (3), Kenya (2), Mexico (2)	Adults with COVID-19 who were confirmed by a positive polymerase-chain-reaction test and radiographic imaging	249 randomly allocated to tocilizumab and 128 to placebo	Tocilizumab was given intravenously as one or two doses of 8 mg per kilogram of body weight (maximum of 800 mg)	Tocilizumab was superior to placebo in the cumulative percentage of receiving mechanical ventilation or death by day 28 (12.0% vs. 19.3%; HR, 0.56; 95% CI, 0.33 to 0.97); there was no significant difference in death from any cause by day 28 (10.4% vs. 8.6%)	Serious adverse events occurred in 38 of 250 patients (15.2%) in the tocilizumab group and 25 of 127 patients (19.7%) in the placebo group
Tocilizumab	COVACTA [23]	The United States (23 sites), France (7), Spain (7), the United Kingdom (7), Canada (4), Denmark (4), Germany (4), Netherlands (4), Italy (2)	Adults with COVID-19 who had blood oxygen saturation of 93% or less or a ratio of the arterial oxygen partial pressure to fractional inspired oxygen of less than 300 mmHg	294 randomly allocated to tocilizumab and 144 to placebo	Tocilizumab was given intravenously as one a dose of 8 mg per kilogram of body weight, followed by either a second dose 8–24 h later or not	The use of tocilizumab did not result in significantly better clinical status at 28 days; mortality at day 28 was 19.7% in the tocilizumab group and 19.4% in the placebo group	Serious adverse events occurred in 103 of 295 patients (34.9%) in the tocilizumab group and in 55 of 143 patients (38.5%) in the placebo group
Tocilizumab	REMDACTA [24]	The United States (41 sites), Spain (9), Brazil (8), Russian Federation (5)	Patients with COVID-19 requiring more than 6 L/min supplemental oxygen to maintain oxygen saturation >93% who received remdesivir (10 days) and either tocilizumab (1 day) or placebo (control)	649 enrolled	Tocilizumab was given intravenously as one dose	Ongoing	Ongoing

Abbreviations: CI, confidence interval; COVID-19, coronavirus disease 2019; HR, hazard ratio; RR, risk ratio.

**Table 3:** The summary of available drugs for the treatment of moderate-to-severe COVID-19.

Drugs	Possible Mechanisms	Indications for the Treatment of COVID-19	Routes of Administration	Recommended Dosage	Related Adverse Events
Remdesivir	Nucleotide analogue; inhibit viral nucleotide synthesis to stop viral replication.	Approved by the US FDA in hospitalized patients aged ≥12 years and weighing ≥40 kg; granted an EUA by the US FDA in hospitalized patients aged <12 years and weighing ≥3.5 kg.	Intravenous.	A single loading dose of 200 mg followed by once-daily maintenance doses of 100 mg for 5 to 10 days or until hospital discharge.	Alterations in liver function studies, increased serum levels of creatinine, atrioventricular block, and anaphylaxis.
Baricitinib	JAK inhibitor; blocks JAK-STAT signaling pathway and inflammatory response.	Granted an EUA by the US FDA in combination with remdesivir in hospitalized patients aged ≥2 years requiring supplemental oxygen, invasive mechanical ventilation, or extracorporeal membrane oxygenation.	Oral.	4 mg in patients aged over 9 years and 2 mg aged under 9 years once daily for 14 days or until hospital discharge.	Thromboembolism, secondary infections, and hypersensitivity reaction.
Tocilizumab	IL-6 receptor antagonist; inhibit IL-6 signaling pathway.	Granted an EUA by the US FDA in hospitalized patients aged ≥2 years receiving systemic corticosteroids and requiring supplemental oxygen, non-invasive or invasive mechanical ventilation, or extracorporeal membrane oxygenation.	Intravenous.	First dose: a single loading dose of 8 mg/kg in patients over 30 kg, and 12 mg/kg in patients under 30 kg (max. 800 mg). Second dose: another single loading dose at least 8 h later if not improved.	Alteration in liver function studies, neutropenia, secondary infections, and allergic reactions.
Sarilumab	IL-6 receptor antagonist; inhibit IL-6 signaling pathway.	Under investigational use.	Subcutaneous or intravenous.	A single loading dose of 200 mg subcutaneously or 200 to 800 mg intravenously.	Alteration in liver function studies, neutropenia, secondary infection, and allergic reaction.

Abbreviations: COVID-19, coronavirus disease 2019; EUA, Emergency Use Authorization; IL-6, Interleukin-6; JAK, Janus kinase; STAT, signal transducer and activator of transcription; US FDA, U.S. Food and Drug Administration.

## Data Availability

Data sharing not applicable.
